# Volatile compounds formed during heating of asymmetric distigmasterol-modified acylglycerols as indicators of thermo-oxidative degradation

**DOI:** 10.1038/s41598-025-20536-2

**Published:** 2025-10-17

**Authors:** Anna Grygier, Magdalena Rudzińska, Dominik Kmiecik, Aleksandra Grudniewska

**Affiliations:** 1 Faculty of Food Sciences and Nutrition, Poznań University of Life Sciences, Wojska Polskiego 31, 60-624 Poznań, Poland; 2 Department of Food Chemistry and Biocatalysis, Wrocław University of Environmental and Life Sciences, Norwida 25, 50‑375 Wrocław, Poland

**Keywords:** Structured acylglycerols, Stigmasterol, Volatile compounds, Heating, Degradation, Sterols, Lipids

## Abstract

Phytosterols play a significant role for organisms. They are a component of cell membranes and also have transport functions. They are also important for lower human blood cholesterol levels. The asymmetric distigmasterol-modified acylglycerols (DStA) with oleic and palmitic acid were synthesized as new, more stable derivatives than free phytosterols. The new structure can improve phytosterols solubility in oil. The identification of more stable forms of phytosterols will allow their greater use as a food additive. Assessment of the volatile compounds formed during thermo-oxidative degradation of phytosterols (60 °C and 180 °C), which may affect the flavor of food products, is a rapid and sensitive method for evaluating phytosterol degradation. The GC/MS technique was used for the determination of volatile compounds. The aim of this work was to determine the volatiles formed during storage and thermal degradation of DStA in order to develop a rapid and sensitive system of measuring their degradation. The same compounds were identified for free stigmasterol, its esters with fatty acids, and new DStAs. Eight volatile compounds can act as indicators of sterol degradation during storage and heating. The synthesized 2,3-distigmasterylsuccinoyl-1-oleoyl-sn-glycerol (DStS-O) offered the highest thermo-oxidative stability during storage and thermal processing of all the examined acylglycerols.

## Introduction

Sterols play an important role in living organisms. They form components of biological membranes and provide protective, structural, transport, and signaling functions in cells^1^. Sterols of plant origin, referred to as phytosterols, are bioactive compounds that play the same role in plant as cholesterol does in animal cells^2^. Because the chemical structure of phytosterols is very similar to that of cholesterol, they are able to take the place of cholesterol in the human blood and reduce its total and LDL fractions^3^. In human organisms after hydrolyzation free phytosterols are absorbed into enterocytes by ATP-binding cassette transporters. A daily supply of 2–3 g of phytosterols will reduce LDL-cholesterol by about 10%–15%^4^. Reduced LDL-cholesterol is associated with a reduced risk of developing cardiovascular diseases^5^. The consumption of phytosterols upsets the production of carcinogens, the growth of cancer cells, angiogenesis, and promotes apoptosis of cancer cells^6^. These factors also diminish the risk of lung, stomach, ovarian, and breast cancer^7^. Phytosterols are involved in the regulation of lipid metabolism and may affect factors that play a role in dementia in the elderly^8^.

The main sources of phytosterols in the human diet are vegetable oils, nuts, cereals, and vegetables. The highest content of phytosterols is found in corn oil, reaching up to 1300 mg per 100 g of oil. Consumption of these products rarely allows 2–3 g of phytosterols per day in the daily diet. Therefore plant sterols are also used as functional additives to food products, such as margarines, yoghurts, juices, and others. Their level in functional foods is much higher than in natural products, reaching as much as 80 mg/g^9^.

Functional products containing phytosterols are often heat-treated during food processing and preparation. An example is margarine with phytosterols, which is often used for frying or baking. The processing temperature can be as high as 200 °C degrees. During that processes phytosterols undergo thermo-oxidative degradation, which may lead to the formation of toxic derivatives^10^. At elevated temperatures and in the presence of oxygen, phytosterols are oxidized to form a variety of oxyphytosterols. The formation of oxyphytosterols may also be influenced by UV radiation and the presence of metallic catalysts. Some of oxyphytosterols are cytotoxic and pro-inflammatory and may increase the risk of cardiovascular diseases and cancer^11,12^.

Fatty acid esters are compounds formed when a fatty acid combines with an alcohol through an esterification reaction. These esters are widely found in nature and have diverse applications, including in foods, cosmetics or biofuels. Sterols exhibit poor solubility in both water and lipids. However, when sterols are esterified with fatty acids, their solubility in fats increases. This enhancement improves their applicability in functional foods. The synthesis of sterol esters is straightforward, and the attachment of a fatty acid moiety does not diminish the biological activity of sterols. The inclusion of phytosterols into glyceride molecules offers greater thermo-oxidative stability^13,14^. The presence of a fatty acid moiety in the fatty acid ester with phytosterol contributes to greater stability of the phytosterol^15^. Additionally, the presence of multiple double bonds in the fatty acid moiety further enhances stability. This is related to competition for oxygen, which first oxidizes the fatty acids and only subsequently the phytosterols. Moreover, the bioavailability of esters is higher compared to free phytosterols. This is presumably due to the fact that gastric acids digest compounds in such a configuration to a lesser extent (in press). To this end, new structured acylglycerols incorporating phytosterols with significantly higher thermo-oxidative stability are being actively sought^14^. To date, the volatile compounds that form during the thermo-oxidation of DStA have not been studied.

Plant sterols and different fatty acids linked by an ester bond with a glycerol backbone have previously been synthesized as more stable alternatives to plant sterols in human nutrition^14,16^. After thermo-oxidative degradation of the asymmetric distigmasterol-modified acylglycerols, low-molecular compounds, which may affect the flavor properties of food products, were identified^14^.

One of the most common phytosterols found in plants is stigmasterol. It contain two double bounds. When stigmasteryl esters with fatty acids are heated, volatile compounds including aldehydes, ketones, alcohols, and hydrocarbons can be identified^13^. Two main compounds, 2-methyl-3-pentanone and 5-ethyl-6-methyl-3-hepten-2-one, have been identified as characteristic degradation products of stigmasterol, and their formation mechanism was elucidated^13^. Both of these volatiles have been suggested as a good indicators of the thermo-oxidative degradation of phytosterols. During heating of distigmasterol-modified acylglycerols at 60 °C and 180 °C, low-molecular compounds were found to constitute 30% and 77% of the nonpolar fraction and 15% and 35% of the polar fraction, respectively^14^. Volatile compounds are part of the low molecular fraction and can be formed during storage and heating of plant oils^17^. They are derivatives mainly of fatty acids, as well as other compounds of oils like sterols or phenolic compounds^18,19^.

One way to determine the degree of degradation of a lipid is to assess the volatile compounds formed during its degradation through headspace analysis of the product. Solid-phase microextraction (SPME) is a quick and environmentally friendly technique (as it uses no solvents) that can characterize the volatiles in the headspace of a product^20^. As a result of identifying the volatile compounds formed during the thermo-oxidation of DStA using the SPME method, it will be possible to rapidly and easily monitor the quality of DStA compounds when incorporated into food products.

The aim of this work was to analyze and identify the components formed during storage and thermal treatment of distigmasterol-modified acylglycerols in order to develop a rapid and sensitive method to assess of degradation.

## Materials and methods

### Materials

Stigmasterol (~ 95%), oleic acid (≥ 99%), palmitic acid (≥ 99%), stigmasteryl chloroformate, dicyclohexyl carbodiimide (DCC), 4-dimethylamino pyridine (DMAP), dichloromethane (≥ 99.8%), hexane (≥ 97%), ethyl acetate (≥ 99.7%), silica gel (70–230 mesh, high purity), pyridine (≥99.8%), succinic anhydride (≥99%), hydrochloric acid (37%), 1-oleoyl-sn-glycerol, 3-palmitoyl-sn-glycerol, magnesium sulfate (≥99.5%), sodium chloride (> 99%), and ethanol-free chloroform were purchased from Sigma-Aldrich (Merck KGaA, Darmstadt, Germany). All the above reagents were used to synthesise acylglycerol esters with phytosterols. Divinylbenzene/carboxene/polydimethylsiloxane (DVB/CAR/PDMS) fiber (50/30 film thickness and 2 cm length) was purchased from Supelco (Merck KGaA, Bellefonte, USA). The fiber was used for SPME analysis.

### Esterification of stigmasterol

The method described in^13^ was used to prepare stigmasterol ester with palmitic acid (St-P) and stigmasterol ester with oleic acid (St-O). Firstly, stigmasterol was dissolved in dichloromethane. The air in a three-necked flask was replaced by argon, and the catalysts (DCC and DMAP) and fatty acids (palmitic or oleic acid) were added and mixed. The reaction proceeded for 24 h in the dark at room temperature (20 °C). The reaction mixture was then washed with water. Further dichloromethane was evaporated and the residue was dissolved in hexane. The resulting mixture was purified on a silica gel column. TLC was used to check the purity of the fractions. Then NMR and GC-MS were used to confirm that stigmasteryl oleate and stigmasteryl palmitate esters had been obtained^13^.

### Synthesis of DStA

DStA was prepared on the basis of^16^. Stigmasterol was covalently attached to 3-palmitoyl-sn-glycerol or 1-oleoyl-sn-glycerol using a succinate or carbonate linker. Stigmasterol derivatization to stigmasterol hemisuccinate was performed first, followed by a Steglich esterification to prepare the 1,2-distigmasterylsuccinoyl-3-palmitoyl-sn-glycerol (DStS-P) and 2,3-distigmasterylsuccinoyl-1-oleoyl-sn-glycerol (DStS-O). 1,2-Distigmasterylcarbonoyl-3-palmitoyl-sn-glycerol (DStC-P) and 2,3-distigmasterylcarbonoyl-1-oleoyl-sn-glycerol (DStC-O) were prepared using 3-acyl-sn-glycerols and stigmasteryl chloroformate.

### Thermo-oxidation of samples

Samples (1 g) of stigmasterol standard, esters of stigmasterol with palmitic acid (St-P) or oleic acid (St-O), 1,2-distigmasteryl-3-palmitoyl glycerols (DStC-P and DStS-P), 2,3-distigmasteryl-1-oleoyl glycerol (DStC-O and DStS-O) were placed in separate glass ampoules (20 ml). The ratio of headspace volume to sample volume was 20. Oxygen was provided in the ampoules to prevent oxygen starvation, and then ampoules were sealed. The samples were heated at 60 °C and 180 °C for eight hours. The temperature of 60 °C is used for the Schaal test, which imitates storage conditions, while 180 °C corresponds to the temperature of frying. After cooling to room temperature, the tip of the vial was cut off and an adapter was applied for SPME analysis. Samples were analysed immediately. The experiment was performed in two replicates.

### Analysis of volatile compounds by SPME-GC-MS

All the samples kept at 60 °C and 180 °C, along with the unheated samples, were analysed. To this end, we used headspace solid-phase micro-extraction (SPME) using DVB/CAR/PDMS fiber. Before analyzing the first sample the fiber was conditioned in the GC injection port at 270 °C for 4 h. Then fiber was placed in the vial with the sample using the adapter for 5 min at room temperature. After that, the fiber was put into the injection port of a gas chromatograph for desorption, which lasted 5 min at 260 °C in splitless mode. A 7890 A GC system (Agilent Technologies, Santa Clara, United States) coupled to a 5975 C VL Triple-Axis mass detector (Agilent Technologies, Santa Clara, United States) was used for the analysis. Separation was run on a DB-5MS capillary column (25 m × 0.2 mm; 0.33 μm film thickness; J&W, Folsom, California) with helium as the carrier gas at a flow rate of 0.6 mL/min. The temperature of the injector and transfer line were 260 °C and 280 °C, respectively. The oven temperature program was as follows: the initial temperature of 40 °C was held for 3 min, then increased at 4 °C/min to 160 °C, and further increased at 10 °C/min to 280 °C; the final temperature was held for 3 min. Masses were scanned from 33 to 333 Da. The ionization energy value was set to 70 eV. Retention indices were calculated for each compound using a homologous series of C7–C24 n-alkanes^21^. NIST library 05 was used to identify volatiles, which were compared with retention indices (RI) from the literature^22^.

### Statistical analysis

Statistical analysis was carried out using Statistica (version 13.3, Tibco Software, Palo Alto, CA, USA). One-way analysis of variance (ANOVA) was followed by Tukey’s *post hoc* test to determine the differences in mean values of volatile peak area amount in the different new compounds of DStA. Student’s *t*-test was used to determine the significant difference between two independent groups. Equality of variance was verified using Levene’s test.

## Results and discussion

### Quantity analysis

**Fig. 1:**
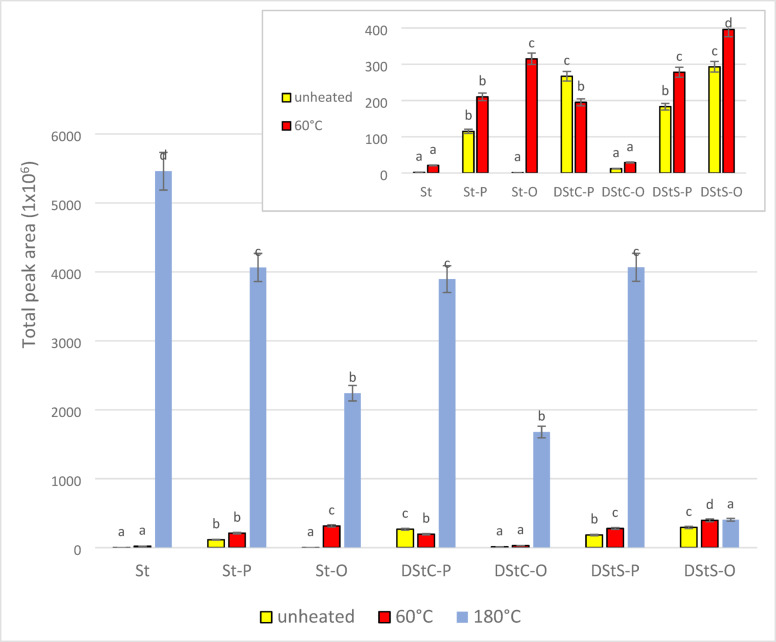
Comparison of the total peak areas of volatile compounds formed during heating of compounds at 60 °C and 180 °C for 8 h. St: stigmasterol; St-P: stigmasteryl palmitate; St-O: stigmasteryl oleate; DStC-P: 1,2-distigmasterylcarbonoyl-3-palmitoyl-*sn*-glycerol; DStC-O: 2,3-distigmasterylcarbonoyl-1-oleoyl-*sn*-glycerol; DStS-P: 1,2-distigmasterylsuccinoyl-3-palmitoyl-*sn*-glycerol; DStS-O: 2,3-distigmasterylsuccinoyl-1-oleoyl-*sn*-glycerol. ^a,b,c…^ -values marked with the same letter at the same temperature do not differ statistically at the significance level α = 0.05.

The volatile compounds formed during thermo-oxidation of DStA at 60 °C and 180 °C were determined using SPME and identified by GC-MS. Stigmasterol standard and esters with palmitic and oleic acids were used as controls. Figure 1 presents the total peak areas of volatiles formed before and after thermo-oxidation for all the compounds. The lowest peak area of the volatile compounds was recorded for the unheated samples, and ranged from 1.0 × 10^6^ for St-O to 3.0 × 10^8^ for DStS-O and DStC-P. It confirm the stability of these samples in the absence of heat. The total peak area was statistically similar for St-O, St, and DStC-O (ranging from 1.0 × 10^6^ to 1.2 × 10^7^) and differed significantly for St-P and DStS-P, at 1.2 × 10^8^ and 1.8 × 10^8^, respectively.

In the samples heated at 60 °C, the total peak area ranged from 2.1 × 10^7^ to 4.0 × 10^8^ and again was lowest for St and DStC-O and highest for DStS-O. For all other samples, the total peak areas were similar at about 2.0 to 3.0 × 10^8^. For DStC-P heated at 60 °C, a decrease was observed in the total peak area, while other samples showed an increase. A twenty-fold increase was found for heated free St. This confirms the low stability of free St during storage.

The samples heated at 180 °C formed much higher total peak areas than those heated at 60 °C. Free St was had the largest peak areas (5.5 × 10^9^). The total peak area for samples with oleic acid ranged from 4.1 × 10^8^ for DStS-O to 2.0 × 10^9^ for St-O and DStC-O, and was much higher (at 4.0 × 10^9^) for samples with palmitic acid (St-P, DStC-P, DStS-P). This is consistent with the results of^13^, where the sum of the peak areas was higher for thermal oxidation of a saturated acid (stearic acid) than of an unsaturated acid (oleic acid) at 60 °C and 180 °C. Comparing the results after heating at 180 °C, the presence of oleic acid that has the greatest influence on the stability of the compound and the formation of volatile compounds. Both St-O and DStC-O and DStS-O have lower values compared to their palmitic acid counterparts. In the case of esters of phytosterols and fatty acids, the presence of unsaturated fatty acids has a stabilizing effect on the phytosterols.

The smallest difference in total peak area between unheated samples and samples heated to 180 °C was found for DStS-O, although the unheated compound had the largest total peak area of all the compounds without heating. It is possible that during the synthesis of this compound, transformations take place affecting the formation of volatile compounds, while during storage as well as heating at 180 °C the compound is stable. Unheated free St had the lowest total peak area for volatiles and the highest total peak area upon heating it to 180 °C. The lowest total peak area is due to the fact that it is a pure single compound and not two compounds linked by an ester bond. Free stigmasterol was not chemically reacted unlike the other compounds. Therefore, free St has the smallest peak area of the volatile compounds. Due to the lack of a St-fatty acid linkage, it is the least stable compound of all the compounds tested and therefore the peak area of the volatile compounds is the highest.

The total peak area of most samples heated at 60 °C was higher than that of the unheated samples, although the opposite held in the case of DStC-P. The smallest increase in the total peak area was found when DStS-O was heated to 60–180 °C. This means that both the linker (hemisuccinate) used to attach the stigmasterol and the fatty acid (oleic acid) attached to the acylglycerol molecule are the best for the stability of this compound. The stability of the compound does not depend on the type of bond linking the phytosterol to the fatty acid, but rather on the number of double bonds present in the fatty acid. These results are similar to those of previous research results that indicated the DStS-O compound to have the highest oxidative stability^14,15^.

### Volatiles formed during heating of St, St-P, and St-O

The volatiles formed by stigmasterol and its esters differ not only in terms of their peak areas, but also in their composition. Table 1 shows the chemical identity and percentages of volatile compounds formed during heating of stigmasterol and stigmasteryl esters with palmitic and oleic acids. The unheated stigmasterol standard had only three volatile compounds: 2-methyl-3-pentanone, nonane and tetradecane (42%, 31%, 28% respectively). Under unheated conditions, stigmasterol undergoes minimal degradation or oxidation. It suggest that these volatiles may arise from minor, spontaneous autoxidation or residual impurities rather than from significant thermal or oxidative breakdown of the sterol structure. This confirms the overall chemical stability of free stigmasterol in the absence of thermal stress. Four volatiles were found when cholesterol degraded: dodecane, tetradecane, acetic acid and hexanal, which were observed at concentrations of 82%, 13%, 3%, and 2%, respectively^23^.

**Table 1:** Percentage peak area (% ± SD) of volatile compounds formed during heating of stigmasterol (St) and stigmasterol esters (St-P and St-O) at 60 °C and 180 °C (8 h).

Volatiles	RI	Unheated			Heated at 60 °C			Heated at 180 °C		
		St	St-P	St-O	St	St-P	St-O	St	St-P	St-O
*Hydrocarbons*										
2-Methylpentane	559						2 ± 0.1^b^			1 ± 0.1^a^
**3-Methylpentane** ^*^	579						2 ± 0.1			
**Heptane**	700						6 ± 0.4^a^			4 ± 0.3^a^
**3-Ethylheptane**	871								5 ± 0.3	
**Nonane**	900	31 ± 2.1								
**Decane**	1000						1 ± 0.1^a^		2 ± 0.2^a^	
**2-Methyldecane**	1061						4 ± 0.3			
**Undecane**	1100		2 ± 0.1^a^	100 ± 5.5^d^		3 ± 0.2^b^	20 ± 1.2^c^		2 ± 0.1^a^	
**Tetradecane**	1400	28 ± 1.1^c^	1 ± 0.2^b^			1 ± 0.1^a^			0 ± 0.1^a^	
*Alcohols*										
**2-Propanol**	515				81 ± 4.3					
**3-Hexanol**	779		7.±0.3^a^			15 ± 0.1^b^				
2-Methyl-3-pentanol^#^	789							4 ± 0.3^b^	4 ± 0.2^b^	2 ± 0.1^a^
**2-Hexanol**	801		7 ± 0.3^a^			14 ± 0.8^b^				
2,2-Diethyl-1,3-propanediol	1118								4 ± 0.3	
*Aldehydes*										
**2-Butenal**	651							1 ± 0.1		
**2-Pentenal** ^**#**^	754						1 ± 0.1^a^	12 ± 0.9^c^	10 ± 0.8^c^	6 ± 0.3^b^
**4-Methyl-3-pentenal** ^**#**^	791							7 ± 0.5^c^	4 ± 0.3^b^	2 ± 0.2^a^
**Hexanal**	800								2 ± 0.1	
2-Ethyl-3-methyl-butanal^#^	847				5 ± 0.3^b^			7 ± 0.6^d^	6 ± 0.5^c^	2 ± 0.1^a^
2-Methyl-2-hexenal	891							2 ± 0.2^a^	2 ± 0.1^a^	
**Heptanal**	901		2 ± 0.1^a^			4 ± 0.2^c^	3 ± 0.1^a^		3 ± 0.2^b^	5 ± 0.3^c^
3,3-Dimethylhexanal	915							5 ± 0.3		
**Octanal**	1004								2 ± 0.1^a^	6 ± 0.4^b^
**2-Ethylhexenal** ^**#**^	1011							9 ± 0.7^c^	3 ± 0.1^b^	2 ± 0.1^a^
**Nonanal**	1106		9 ± 0.3^d^			9 ± 0.5^d^	4 ± 0.2^b^		1 ± 0.1^a^	7 ± 0.4^c^
**Decanal**	1207		2 ± 0.1^c^			1 ± 0.1^b^			0.±0.1^a^	
**2-Decenal**	1250						2 ± 0.1^a^			1 ± 0.1^a^
*Ketones*										
**Acetone**	511									4 ± 0.3
**2-Methyl-3-pentanone** ^**#**^	765	42 ± 2.3^c^			6 ± 2.1^a^			7 ± 0.5^a^	10 ± 0.8^b^	7 ± 0.4^a^
**3-Hexanone**	784		13 ± 0.8^c^			13 ± 0.9^c^	1 ± 0.1^b^			0 ± 0.1^a^
**2-Hexanone**	803		11 ± 0.8^b^			11 ± 0.7^b^	1 ± 0.1^a^		2 ± 0.1^a^	2 ± 0.1^a^
**2-Heptanone**	889						1 ± 0.1^a^			4 ± 0.2^b^
**Cyclohexanone**	894		30 ± 1.2^b^			25 ± 1.7^a^	27 ± 1.9^a^			
**2-Methylcyclohexanone**	937						8 ± 0.6			
**2-Octanone**	988						1 ± 0.1^a^		2 ± 0.1^a^	3.±0.2^b^
3-Methyl-3-cyclohexen-1-one	1010							2 ± 0.2^a^	3 ± 0.2^b^	2 ± 0.1^a^
**2-Nonanone**	1091						2 ± 0.1^a^			1 ± 0.1^a^
5-Ethyl-6-methyl-3-hepten-2-one^#^	1143				3 ± 0.2^a^			24 ± 1.8^c^	13 ± 1.0^b^	11 ± 0.8^b^
*Acids*										
**Octanoic Acid**	1212		2 ± 0.2^c^			1 ± 0.1^b^			1 ± 0.1^a^	1 ± 0.1^b^
**Nonanoic acid**	1267		1 ± 0.1^c^			1 ± 0.1^b^			0 ± 0.1^a^	
*Others*										
			11 ± 0.7^c^		4 ± 0.2^b^	2 ± 0.2^a^	15 ± 1.1^d^	20 ± 1.3^e^	18 ± 1.2^e^	28 ± 1.4^f^

^*^: Volatile compounds which possess an odor are shown in bold (based on https://pubchem.ncbi.nlm.nih.gov); ^#^: compounds detected in samples heated at 180 °C; ^a, b,c...^ - values marked with the same letter in a rows do not differ statistically at the significance level α = 0.05; St: stigmasterol; St-P: stigmasteryl palmitate; St-O: stigmasteryl oleate.

The dominant volatile in free stigmasterol heated to 60 °C was 2-propanol, which make up 81% of total volatiles. The greatest contribution in the case of St-P, unheated and heated at 60 °C, was made by cyclohexanone from stigmasterol structure at 30% and 25% respectively. Only undecane (100%) from fatty acid chain was detected in unheated St-O, while cyclohexanone (27%) was the main compound when this was heated at 60 °C.

**Fig. 2:**
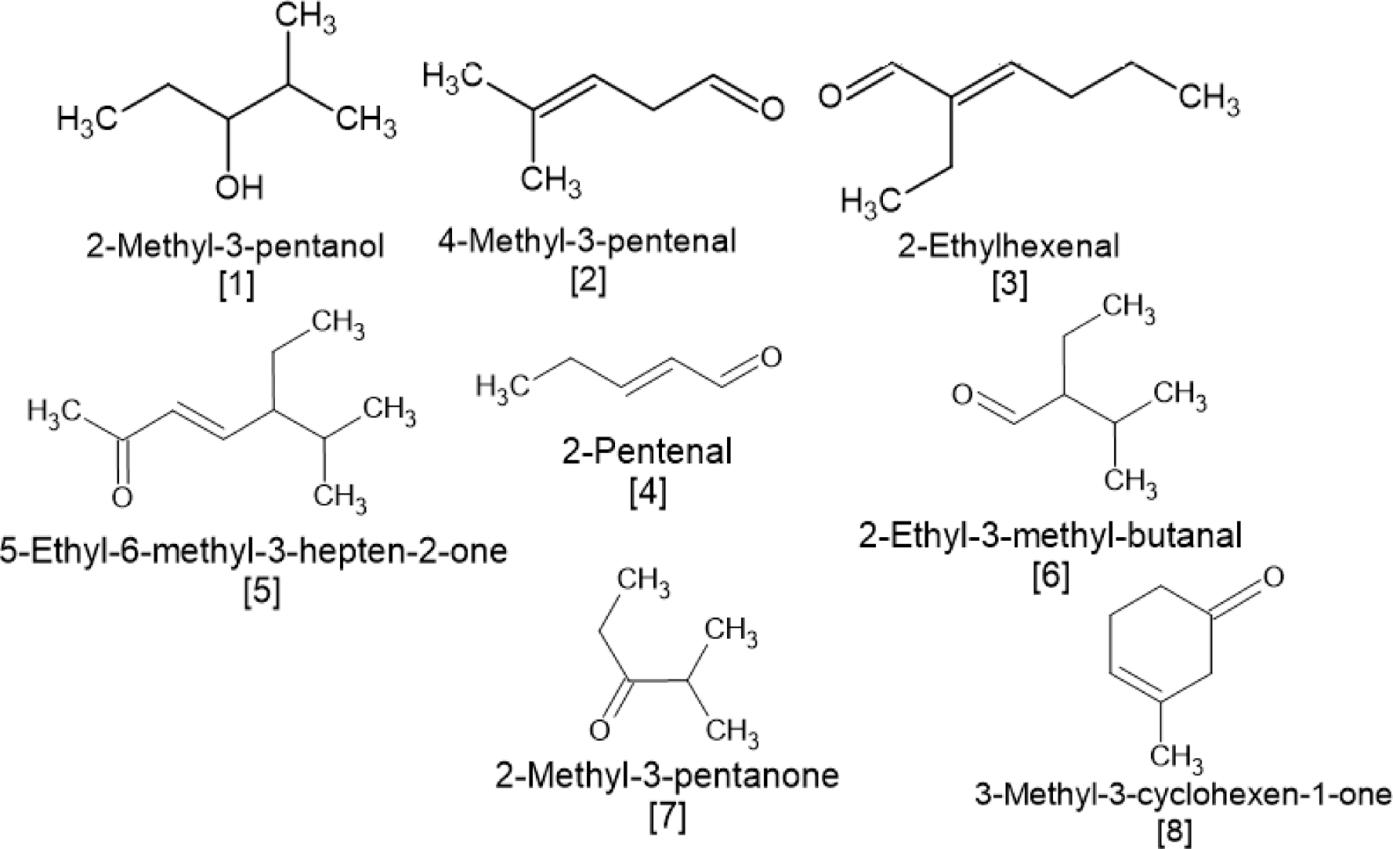
Chemical structure of main volatile compounds formed during heating of stigmasterol and stigmasterol esters (St, St-P, St-O) at 180 °C.

When stigmasterol and its esters were heated at 180 °C, eight compounds were detected in all samples, including 2-methyl-3-pentanol [1], 4-methyl-3-pentanal [2], 2-ethylhexenal [3], 2-pentenal [4], 5-ethyl-6-methyl-3-hepten-2-one [5], 2-ethyl-3-methyl-butanal [6], 2-methyl-3-pentanone [7] and 3-methyl-3-cyclohexen-1-one [8] (Fig. 2). Two of these compounds most likely originated from the degradation of stigmasterol, namely: 2-ethyl-3-methyl-butanal and 2-pentenal. These compounds are formed as a result of side chain cleavage.

**Fig. 3:**
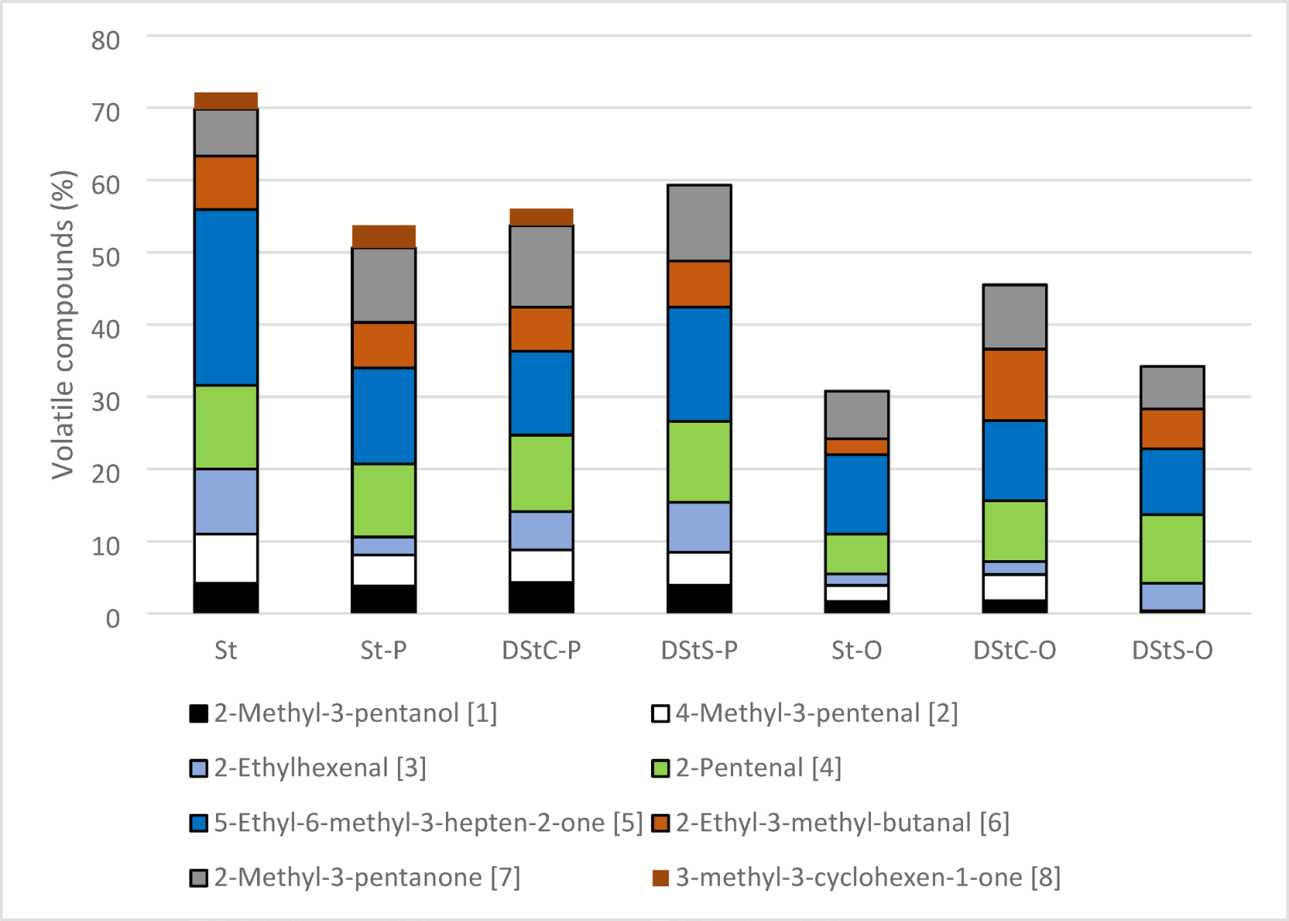
Volatile compounds typical of stigmasterol (St), stigmasteryl palmitate (St-P), stigmasteryl oleate (St-O), 1,2-distigmasterylcarbonoyl-3-palmitoyl-sn-glycerol (DStC-P), 2,3-distigmasterylcarbonoyl-1-oleoyl-sn-glycerol (DStC-O), 1,2-distigmasterylsuccinoyl-3-palmitoyl-sn-glycerol (DStS-P), and 2,3-distigmasterylsuccinoyl-1-oleoyl-sn-glycerol (DStS-O) heated at 180 °C.

In heated stigmasterol the predominant compound was 5-ethyl-6-methyl-3-hepten-2-one, which accounted for 24% and from 11% to 13% for St-O and St-P, respectively (Fig. 3). For all these compounds other than 2-methyl-3-pentanone [7] and 3-methyl-3-cyclohexen-1-one [8] the highest proportion was found in heated stigmasterol, lower levels were noted in St-P, and the lowest levels were in St-O. This may be due to the protective effect of fatty acids during thermal oxidation of stigmasterol, which was also observed by^15^.

Heptane, heptanal, octanal, nonanal, 2-decanal, and octanoic acid were formed from the decomposition of oleic acid and decane, undecane, dodecane, tridecane, tetradecane, hexanal, octanal, nonanal, decanal, 2-heptanone, 2-hexanone, and 2-octanone derived from the decomposition of palmitic acid^13,24^.

### Volatiles formed during heating of DStA

The volatile compounds determined in DStA before and after heating at 60 °C and 180 °C for eight hours are shown in Table 2. In freshly prepared DStA, the number of volatiles ranged from one compound (decane) for DStC-O to seven compounds (decane, undecane, 1-dodecene, dodecane, tridecane, 1-tetradecene, oleic acid) for DStS-O. The main volatile compound identified in all the unheated samples was decane, whose proportion ranged from 42% to 100% in DStS-O and DStC-O, respectively. Decane, which was also detected in esters held at 180 °C, was probably formed from fatty acids, rather than being a derivative of sterol.

**Table 2:** Percentage peak area (% ± SD) of volatile compounds from unheated DStA and from heating DStA at 60 °C and 180 °C (8 h).

Volatiles	RI	DStC-P			DStC-O				DStS-P				DStS-O		
Heating temperatures		0 °C	60 °C	180 °C	0 °C	60 °C	180 °C	0 °C		60 °C	180 °C	0 °C		60 °C	180 °C
*Hydrocarbons*															
Heptane*	700														3 ± 0.2
**Octane**	800						4 ± 0.3^a^								7 ± 0.5^b^
**Nonane**	900		3 ± 0.2^b^	2 ± 0.4^a^						2 ± 0.5^a^					
**1-Decene**	986						2 ± 0.4^b^			1 ± 0.3^a^				1 ± 0.1^a^	4 ± 0.7^c^
**Decane**	1000	53 ± 2.1^e^	67 ± 4.5^g^	5 ± 0.3^a^	100 ± 0.3	32 ± 2.4^c^		50 ± 3.7^e^		58 ± 3.6^f^	4 ± 0.2^a^	42 ± 2.1^d^		30 ± 1.8^c^	10 ± 0.7^b^
**Undecane**	1100	9 ± 0.5^c^	11 ± 0.4^d^	3 ± 0.1^a^				8 ± 0.5^c^		11 ± 0.8^d^	3 ± 0.1^a^	16 ± 0.9^e^		11 ± 0.7^d^	4 ± 0.3^b^
3-Dodecene	1185					8 ± 0.6									
**1-Dodecene**	1191									3 ± 0.2^b^		1 ± 0.1^a^		9 ± 0.6^c^	
**Dodecane**	1200	4 ± 0.2^c^	5 ± 0.2^d^	2 ± 0.1^a^		6 ± 0.4^e^		6 ± 0.3^e^		6 ± 0.4^f^	2 ± 0.1^a^	7 ± 0.5^f^			3 ± 0.1^b^
**Tridecane**	1300	4 ± 0.3^b^	4 ± 0.1^b^	1 ± 0.1^a^		12 ± 0.9^d^	0 ± 0.1^a^	7 ± 0.5^c^		7 ± 0.4^c^	1 ± 0.1^a^	18 ± 1.1^f^		15 ± 0.9^e^	7 ± 0.5^c^
**1-Tetradecene**	1391									1 ± 0.1^a^		3 ± 0.1^b^		3 ± 0.2^b^	1 ± 0.1^a^
3-Methyleicosane	2072					9 ± 0.6									
*Alcohols*															
3-Methyl-2-pentanol	762													16 ± 0.9	
2-Methyl-3-pentanol^#^	789			4 ± 0.3^c^			2 ± 0.1^b^				4 ± 0.3^c^				0 ± 0.1^a^
*Aldehydes*															
**2-Pentenal** ^**#**^	754			11 ± 0.7^c^			8 ± 0.6^a^				11 ± 0.9^c^				10 ± 0.8^b^
**4-Methyl-3-pentenal** ^**#**^	791			5 ± 0.3^b^			4 ± 0.3^a^				5 ± 0.3^b^				
2-Ethyl-3-methyl-butanal^#^	847			6 ± 0.5^b^		8 ± 0.6^c^	10 ± 0.6^d^				6 ± 0.5^b^				6 ± 0.2^a^
2-Methyl-2-hexenal	891			2 ± 0.1^b^			1 ± 0.1^a^				1 ± 0.1^a^				
**Heptanal**	901			3 ± 0.1^a^			7 ± 0.4^c^				3 ± 0.1^a^				4 ± 0.2^b^
3,3-Dimethylhexanal	915			4 ± 0.2^a^			4 ± 0.2^a^								
**Octanal**	1004			1 ± 0.1^a^			9 ± 0.6^d^				2 ± 0.2^a^				6 ± 0.4^b^
**2-Ethylhexenal** ^**#**^	1011			5 ± 0.3^c^			2 ± 0.1^a^				7 ± 0.5^d^				4 ± 0.3^b^
**Nonanal**	1106						9 ± 0.6^c^				1 ± 0.1^a^				7 ± 0.3^b^
**2-Decenal**	1250						1 ± 0.1^a^								2 ± 0.1^b^
*Ketones*															
**Aceton**	511	29 ± 1.8^c^						25 ± 2.0^b^			2 ± 0.1^a^				
**2-Methyl-3-pentanone** ^**#**^	765			11 ± 0.7^c^		13 ± 0.9^d^	9 ± 0.5^b^				11 ± 0.7^c^				6 ± 0.4^a^
3-Hydroxy-3-methyl-2-butanone	779					13 ± 0.8^b^								0.6 ± 0.1^a^	
**2-Octanone**	988			1 ± 0.2^a^			1 ± 0.1^a^				2 ± 0.4^b^				
**2-Nonanone**	1091						1 ± 0.1^a^				1 ± 0.1^a^				
5-Ethyl-6-methyl-3-hepten-2-one^#^	1143			12 ± 0.9^b^			11 ± 0.6^b^				16 ± 0.8^c^				9 ± 0.6^a^
*Acids*															
**Oleic acid**	2115											10 ± 0.7			
*Others*															
		2 ± 0.1^a^	8 ± 0.5^d^	19 ± 1.1^g^			11 ± 0.9^f^	3 ± 0.1^b^		9 ± 0.5^e^	19 ± 1.2^g^	3 ± 0.8^b^		9 ± 0.6^e^	6 ± 0.4^c^

*: Volatile compounds which possess an odor are shown in bold (based on https://pubchem.ncbi.nlm.nih.gov); ^#^: compounds detected in samples heated at 180 °C; ^a, b,c…^ - values marked with the same letter in a rows do not differ statistically at the significance level α = 0.05.

DStA: distigmasterol-modified acylglycerols; DStC-P:1,2-distigmasterylcarbonoyl-3-palmitoyl-*sn*-glycerol; DStC-O: distigmasterylcarbonoyl-1-oleoyl-*sn*-glycerol; DStS-P: 1,2-distigmasterylsuccinoyl-3-palmitoyl-*sn*-glycerol; DStS-O: 2,3-distigmasterylsuccinoyl-1-oleoyl-*sn*-glycerol.

The degradation of DStA during heating at 60 °C was slow, and oxidative derivatives were formed rather than thermal degradation products. In DStA with palmitic acid molecules, the percentage of decane increased during heating at 60 °C from 50% to 53% to 58%–67%. The opposite was the case in samples with oleic acid: decane decreased from 42% to 100% to 30%–32%. This may be due to the merging of the three decane molecules and the formation of tridecane, because the percentage of tridecane increased in these samples.

Distigmasterol-modified acylglycerols with palmitic acid heated to 60 °C only formed volatile chain hydrocarbons with six to fourteen carbon atoms. When oleic acid replaced palmitic acid, ketones, aldehydes, and alcohols were detected alongside hydrocarbons (Table 2). The presence of a double bond in fatty acids esterified with sterols and distigmasterol-modified acylglycerols affects positively on the thermo-oxidative stability of sterol part^14^.

During heating of DStA at 180 °C, much greater levels of volatile compounds were produced than at 60 °C. In all the DStA, the same seven volatiles that were noted for St, St-P and St-O heated at 180 °C were detected, along with 3-methyl-3-cyclohexen-1-one [8] and 4-methyl-3-pentenal [2] (Tables 1 and 2). The chemical structures of these compounds are presented in Fig. 2.

The percentage composition of volatile compounds depended on the fatty acid molecule bonded to glycerol and on the linker bonded to stigmasterol and glycerol. The highest total percentage of the seven main volatiles was determined for DStS-P and made up 59% of all determined volatiles (Fig. 3). The main component was 2-methyl-3-pentanone [7], which made up 16%, then 4-methyl-3-pentenal [2], 2-ethyl-3-methyl-butanal [6], 5-ethyl-6-methyl-3-hepten-2-one [5], 2-pentenal [4], 2-ehylhexenal [3] and 2-methyl-3-pantanol [1] which respectively made up 11%, 10%, 7%, 6%, 5%, and 4% (Table 2).

For DStC-P, the most common volatile was also 2-methyl-3-pentanone [7], followed by 2-ethyl-3-methyl-butanal [6], 4-methyl-3-pentenal [2], 2-pentenal [4], 2-ethylhexenal [3], 5-ethyl-6-methyl-3-hepten-2-one [5], and 2-methyl-3-pantanol [1]; in total these made up 54% (Table 2).

When DStC-O was held at 180 °C, 2-methyl-3-pentanone [7] was again the most common volatile, but 2-pentenal [4], 2-ethyl-3-methyl-butanal [6], and 4-methyl-3-pentenal [2] were nearly as common, while 2-ethylhexenal [3], 5-ethyl-6-methyl-3-hepten-2-one [6], and 2-methyl-3-pantanol [1] were much less frequent. These compounds constituted 46%.

Volatile compounds were least common for DStS-O, where they made up 34%. 4-methyl-3-pentenal [2] and 2-methyl-3-pentanone [7] dominated, followed by 2-ethyl-3-methyl-butanal [6], 2-pentenal [4], and 5-ethyl-6-methyl-3-hepten-2-one [5]. The percentage of 2-methyl-3-pantanol [1] and 2-ethylhexenal [3] was below 0.5%.

2-methyl-3-pentanone [7], 2-ethyl-3-methyl-butanal [6] and 4-methyl-3-pentenal [2] were the most common volatiles of all samples. The compounds 5-ethyl-6-methyl-3-hepten-2-one [5] and 2-methyl-3-pentanone [7] have also been identified by ^13^. Hypothetical routes of formation for 5-ethyl-6-methyl-3-hepten-2-one and 2-methyl-3-pentanone have been presented by ^13^. Both compounds were created through the autoxidized side chain of stigmasterol. These volatiles have been suggested as indicators for the degradation of phytosterols or its esters during heating of food products.

Other compounds, such as 4-methyl-3-pentenal [2], 2-pentanal [4] and 2-ethylhexenal [3], have been identified as volatile derivatives formed during the thermal processing of plant oils and fatty acid^25,26^. 2-ethylhexenal [3] has been detected in naked and covered oat groats and flakes and in the head of insects, where it has also been suggested as a derivative of fatty acids^27,28^, though this suggestion probably needs correcting. Volatile compounds formed during thermal processing may also originate from other components found in fats and cooking oils, such as sterols, tocopherols, or phenolic compounds.

Our results allowed us to detail and extend the range of volatile compounds formed during the processing and storage of acylglycerols containing phytosterols. For the first time, eight volatile compounds have been identified that can affect the aroma of these foods, regardless of the form in which the plant sterols are present or have been added to the food.

The thermo-oxidative stability, cytotoxicity, and genotoxicity of the distigmasterol-modified acylglycerols (DStA) have been assessed in our previous publications^14,16^. The aim of the study was to determine the properties of new stigmasterol structures. Previous study showed that DStS-O was the most stable compound during heating and storage and showed no cytotoxic or genotoxic properties. These compounds have been synthesized for the first time, and a rapid and convenient method for determining their stability during storage and heating should be developed before they are used in functional foods; analyzing the volatile compounds formed during their degradation seems to meet these conditions. We thus attempted to identify the key compounds responsible for the negative transformation of DStA.

The formation of volatile compounds during thermo-oxidation is influenced by multiple processes. The principal mechanisms occurring in oil systems include autoxidation, photo-oxidation, thermo-oxidation, and enzymatic oxidation. All of these pathways lead to the degradation of lipid hydroperoxides, whose decomposition represents the key source of volatile compounds responsible for the characteristic odor and flavor of oils.

Distigmasterol-modified acylglycerols constitute a more complex system compared to conventional oils or pure sterol esters. The mechanisms underlying volatile compound formation in acylglycerol–phytosterol esters include, similarly to oils, the oxidation of fatty acid residues, which is the major source of volatile compounds. In addition, the phytosterol moieties within the molecule may undergo transformation into oxysterols (non-volatile products), which, upon prolonged high-temperature degradation, can further decompose into smaller molecules, some of which are volatile. Moreover, the presence of phytosterols within the ester structure may alter the dynamics of hydroperoxide formation and can either stabilize or destabilize lipid free radicals. This indirectly affects the overall volatile profile of the system.

Distigmasterol-modified acylglycerols have the potential to serve as new functional compounds in the enrichment of food products aimed at lowering cholesterol levels in human blood. Phytosterols in the form of esters are likely to be more bioavailable due to the stability of the structure, resulting in less degradation of the compound during digestion. Eight volatile compounds selected from free stigmasterol, its esters, and acylglycerols can act as indicators of sterol degradation during storage and heating. The SPME technique for extracting volatile compounds from foods containing phytosterol is a rapid and convenient method for detecting degradation. Our study has additionally shown that oleic acid bonded with stigmasterol as ester or acylglycerol protects stigmasterol against thermo-oxidative degradation. This has the potential to serve as a safer source of phytosterols in the human diet while allowing rapid detection of its degradation in food products. Further research should address the absorption of this compound in the human gastrointestinal tract, as the use of two stigmasterol molecules may make it possible to decrease the dose needed to lower cholesterol levels.

## Data Availability

The datasets used and/or analysed during the current study available from the corresponding author on reasonable request.

## Abbreviations

DStA: distigmasterol-modified acylglycerols
DStC-P: 1,2-distigmasterylcarbonoyl-3-palmitoyl-sn-glycerol
DStC-O: 2,3-distigmasterylcarbonoyl-1-oleoyl-sn-glycerol
DStS-P: 1,2-distigmasterylsuccinoyl-3-palmitoyl-sn-glycerol
DStS-O: 2,3-distigmasterylsuccinoyl-1-oleoyl-sn-glycerol
St: stigmasterol
St-P: stigmasterol ester with palmitic acid
St-O: stigmasterol ester with oleic acid

## References

[1] Valitova, J. N., Sulkarnayeva, A. G. & Minibayeva, F. V. Plant sterols: Diversity, Biosynthesis, and physiological functions. *Biochem. (Mosc)*. **81** (8), 819–834. 10.1134/S0006297916080046 (2016). 10.1134/S0006297916080046 27677551

[2] Hemmerlin, A. et al. A review of tobacco BY-2 cells as an excellent system to study the synthesis and function of sterols and other isoprenoids. *Lipids* **39** (8), 723–735. 10.1007/s11745-004-1289-0 (2004). 15638240 10.1007/s11745-004-1289-0

[3] Gupta, A. K., Savopoulos, C. G., Ahuja, J. & Hatzitolios, A. I. Role of phytosterols in Lipid-Lowering: current perspectives. *Eur. J. Lipid Sci. Technol.* **19** (6), 13037–13044. 10.1093/qjmed/hcr007 (2011). 10.1093/qjmed/hcr007 21325285

[4] MacKay, D. S. & Jones, P. J. H. Phytosterols in human nutrition: Type, Formulation, Delivery, and physiological function. *Eur. J. Lipid Sci. Technol.* **113** (12), 1427–1432. 10.1002/EJLT.201100100 (2011).

[5] Plat, J., Strandberg, T. E. & Gylling, H. Intestinal cholesterol and phytosterol absorption and the risk of coronary artery disease. *Eur. Heart J.* 1–2. 10.1093/eurheartj/ehaa881 (2020). 10.1093/eurheartj/ehaa881 33184635

[6] Cioccoloni, G. et al. Phytosterols and phytostanols and the hallmarks of cancer in model organisms: A systematic review and Meta-Analysis. *Crit. Rev. Food Sci. Nutr.* **0** (0), 1–21. 10.1080/10408398.2020.1835820 (2020). 10.1080/10408398.2020.1835820 33238719

[7] Woyengo, T. A., Ramprasath, V. R. & Jones, P. H. J. Anticancer effects of phytosterols. *Eur. J. Clin. Nutr.* **63**, 813–820 (2009). 19491917 10.1038/ejcn.2009.29

[8] Shuang, R., Rui, X. & Wenfang, L. Phytosterols and dementia. *Plant Foods Hum. Nutr.* **71**, 347–354 (2016). 27663717 10.1007/s11130-016-0574-1

[9] Raczyk, M., Bonte, A., Matthäus, B. & Rudzińska, M. Impact of added Phytosteryl/Phytostanyl fatty acid esters on chemical parameters of margarines upon heating and Pan-Frying. *Eur. J. Lipid Sci. Technol.* **120** (2), 1–11. 10.1002/ejlt.201700281 (2018).

[10] Baumgartner, S. et al. Oxyphytosterol formation in humans: identification of high vs. Low oxidizers. *Biochem. Pharmacol.* **86** (1), 19–25. 10.1016/j.bcp.2013.02.035 (2013). 23500537 10.1016/j.bcp.2013.02.035

[11] Kaur, R. & Myrie, S. B. Association of dietary phytosterols with cardiovascular disease biomarkers in humans. *Lipids* **55** (6), 569–584. 10.1002/lipd.12262 (2020). 32557606 10.1002/lipd.12262

[12] Wang, M. & Lu, B. How do oxyphytosterols affect human health? *Trends Food Sci. Technol.* **79**, 148–159 (2018).

[13] Raczyk, M., Kmiecik, D., Przybylski, R. & Rudzińska, M. Effect of fatty acid unsaturation on phytosteryl ester degradation. *J. Am. Oil Chem. Soc.* **94** (5), 701–711. 10.1007/S11746-017-2979-X (2017). 28479606 10.1007/s11746-017-2979-x PMC5397657

[14] Rudzińska, M. et al. Thermo-Oxidative stability of asymmetric Distigmasterol-Modified acylglycerols as novel derivatives of plant sterols. *Food Chem.* **390**, 133150. 10.1016/J.FOODCHEM.2022.133150 (2022). 35551028 10.1016/j.foodchem.2022.133150

[15] Barriuso, B., Astiasarán, I. & Ansorena, D. Unsaturated lipid matrices protect plant sterols from degradation during heating treatment. *Food Chem.* **196**, 451–458. 10.1016/j.foodchem.2015.09.074 (2016). 26593514 10.1016/j.foodchem.2015.09.074

[16] Rudzińska, M. et al. Distigmasterol-Modified acylglycerols as new structured Lipids—Synthesis, identification and cytotoxicity. *Molecules* **26**, 6837 (2021). 34833929 10.3390/molecules26226837 PMC8617691

[17] Xu, L. et al. Comparative analysis of aroma compounds in French Fries and palm oil at three crucial stages by GC/MS-Olfactometry, odor activity Values, and aroma recombination. *J. Sci. Food Agric.* **102** (7), 2792–2804. 10.1002/jsfa.11620 (2022). 34716586 10.1002/jsfa.11620

[18] Genovese, A., Yang, N., Linforth, R., Sacchi, R. & Fisk, I. The role of phenolic compounds on Olive oil aroma release. *Food Res. Int.* **112** (May), 319–327. 10.1016/j.foodres.2018.06.054 (2018). 30131143 10.1016/j.foodres.2018.06.054

[19] Rudzińska, M., Przybylski, R. & Wąsowicz, E. Products formed during Thermo-Oxidative degradation of phytosterols. *JAOCS J. Am. Oil Chemists’ Soc.* **86** (7), 651–662. 10.1007/s11746-009-1397-0 (2009).

[20] Sheehan, E. M., Limmer, M. A., Mayer, P., Karlson, U. G. & Burken, J. G. Time-Weighted average SPME analysis for in planta determination of CVOCs. *Environ. Sci. Technol.* **46** (6), 3319–3325. 10.1021/ES2041898/SUPPL_FILE/ES2041898_SI_001.PDF (2012). 22332592 10.1021/es2041898

[21] van Den Dool, H. & Dec. Kratz, P. A. Generalization of the retention index system including linear temperature programmed Gas—Liquid partition chromatography. *J. Chromatogr. A*. **11** (C), 463–471. 10.1016/S0021-9673(01)80947-X (1963). 10.1016/s0021-9673(01)80947-x 14062605

[22] Adams, R. P. Identification of Essential Oil Components by Gas Chromatograpy/Mass Spectrometry, 4th Edition. *Illinois USA: Allured Publishing Corporation, Carol Stream* 804–806. (2007).

[23] Cardenia, V., Olivero, G. & Rodriguez-Estrada, M. T. Thermal oxidation of cholesterol: preliminary evaluation of 2-Methyl-6-Heptanone and 3-Methylbutanal as volatile oxidation markers. *Steroids* **99** (PB), 161–171. 10.1016/J.STEROIDS.2015.03.017 (2015). 25846978 10.1016/j.steroids.2015.03.017

[24] Liu, X. et al. Effects of low salt on lipid oxidation and Hydrolysis, fatty acids composition and volatiles flavor compounds of Dry-Cured Ham during ripening. *LWT - Food Sci. Technol.* **187**, 115347. 10.1016/j.lwt.2023.115347 (2023).

[25] Zhuang, Y. et al. Impact of heating temperature and fatty acid type on the formation of lipid oxidation products during thermal processing. *Front. Nutr.* **9** (June), 1–10. 10.3389/fnut.2022.913297 (2022). 10.3389/fnut.2022.913297 PMC9201814 35719170

[26] Agelopoulos, N. G. & Keller, M. A. Plant-Natural enemy association in tritrophic System, Cotesia Rubecula-Pieris Rapae-Brassicaceae (Cruciferae). III: collection and identification of plant and Frass volatiles. *J. Chem. Ecol.* **20** (8), 1955–1967. 10.1007/BF02066236 (1994). 24242722 10.1007/BF02066236

[27] Jin, W., Liu, D. & Hu, X. Volatile compounds in Chinese naked and covered oat groats and flakes. *J. Food Process. Preserv*. **42** (8), 1–9. 10.1111/jfpp.13691 (2018).

[28] Voegtle, H. L., Jones, T. H., Davidson, D. W. & Snelling, R. R. E-2-Ethylhexenal, E-2-Ethyl-2-Hexenol, Mellein, and 4-Hydroxymellein in Camponotus species from Brunei. *J. Chem. Ecol.* **34** (2), 215–219. 10.1007/s10886-008-9430-6 (2008). 18213494 10.1007/s10886-008-9430-6

